# 

**Published:** 1891-06

**Authors:** 


*ERRATUM. Selection "Chancres of Fingers" was put in original matter by mistake.

